# Identification of Tiny Surface Cracks in a Rugged Weld by Signal Gradient Algorithm Using the ACFM Technique

**DOI:** 10.3390/s20020380

**Published:** 2020-01-09

**Authors:** Xin’an Yuan, Wei Li, Xiaokang Yin, Guoming Chen, Jianming Zhao, Weiyu Jiang, Jiuhao Ge

**Affiliations:** 1 Center for Offshore Engineering and Safety Technology, China University of Petroleum (East China), Qingdao 266580, China; xinancom@163.com (X.Y.); xiaokang.yin@upc.edu.cn (X.Y.); gmchen@upc.edu.cn (G.C.); jianmingzhao123@163.com (J.Z.); jwy_upc@163.com (W.J.); 2 Nondestructive Detection and Monitoring Technology for High Speed Transportation Facilities, Key Laboratory of Ministry of Industry and Information Technology, Nanjing University of Aeronautics and Astronautics, Nanjing 210016, Jiangsu, China; gejiuhao@163.com

**Keywords:** ACFM, tiny surface crack, rugged weld, signal gradient algorithm

## Abstract

It is still a big challenge to identify tiny surface cracks in a rugged weld due to the lift-off variations using the nondestructive testing (NDT) method. In this paper, the signal gradient algorithm is presented to identify the tiny surface crack in the rugged weld using the alternating current field measurement (ACFM) technique. The ACFM simulation model and testing system was set up to obtain the insensitive signal to the lift-off variations. The signal gradient algorithm was presented to process the insensitive signal for the identification of the tiny surface crack in the rugged weld. The results show that the ***Bz*** signal is the insensitive signal to lift-off variations caused by the rugged weld. The signal to noise ratio (SNR) of the crack identification signal was greatly improved by the signal gradient algorithm, and a tiny surface crack can be identified effectively in the weld and the heat affected zone (HAZ).

## 1. Introduction

The welding procedure is widely used in the manufacturing industry. As a critical connecting portion, it easily introduces cracks in the weld and the HAZ due to the discontinuous material, corrosive environment, varying temperatures, and complex stress. Welds should be inspected routinely using a nondestructive testing (NDT) method in in-service time or before use [1,2,3,4].

Magnetic particle testing (MT) and penetrant testing (PT) are effective methods used to detect surface cracks in a weld. However, the surface of the structure should be cleaned thoroughly, including the coating, the attachment, and the greasy dirt [5,6]. Additionally, the testing result depends on the experience of the operator. Ultrasonic testing (UT) is usually used for the detection of internal defects [7]. Eddy current testing (ET) is easily confused by the lift-off variations, making it hard to identify a tiny crack in a rugged weld [8].

**Figure 1:**
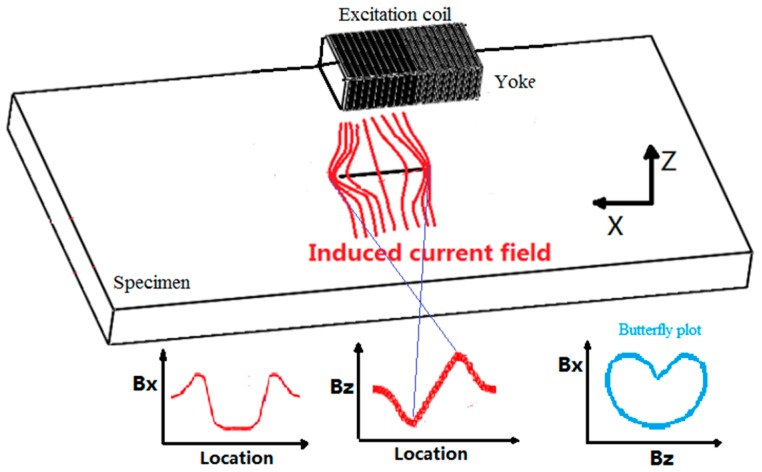
Principle of ACFM.

The ACFM technique was developed for the detection of cracks in underwater structures. Due to the advantages of non-contact testing, less cleaning, and quantitative detection, the ACFM technique has been widely used on land and underwater [9,10,11]. The principle of ACFM is shown in Figure 1. The excitation coil induces the local uniform current field in the specimen. When a crack is present, the current field is disturbed. The disturbed current field makes the space magnetic field distorted. The magnetic field in the X direction, ***Bx*** (parallel to the crack), shows a trough in the center of the crack which contains depth information of the crack. The magnetic field in the Z direction, ***Bz*** (perpendicular to the specimen), shows two opposite peaks at the tips of the crack, which reflects the length of the crack. Normally, a so-called butterfly plot (the ***Bx*** against the ***Bz***) is presented to decide whether a crack is present or not. In practice, the cracks are identified by the number of the loops in the butterfly plot after detection [12].

**Figure 2:**
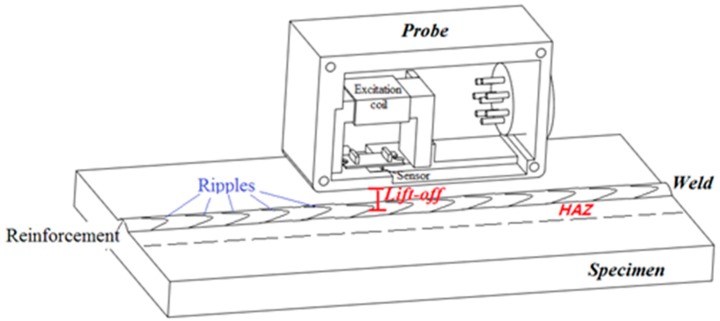
Weld ripples and reinforcement cause lift-off variations.

However, when the ACFM is used to detect tiny cracks in a weld, the ripples and reinforcements can be regarded as the distance variations between the probe and the specimen [13] which causes the lift-off of the probe variations, as shown in Figure 2. The lift-off variations bring many interference signals, making the signal to noise ratio (SNR) poor [14,15]. The response signals of a probe are harsh, even on a crack. The ***Bx*** and ***Bz*** are confused by the noise caused by the lift-off variations. As a result, the conventional butterfly plot cannot effectively identify tiny cracks. Of course, the size of the crack cannot be evaluated by the ***Bx*** and ***Bz*** at this stage. As the first step, how to find a tiny crack in a weld is critical work in the ACFM field. 

The probability of detection (POD) using the ACFM technique decreases drastically for tiny cracks whose lengths are less than 10 mm [16]. Smith et al. applied the ACFM technique to inspect the welds in stainless steel nuclear storage tanks [17]. The results showed that most of the tiny cracks (length less than 10 mm, depth less than 1 mm) could not be identified effectively. Mostafavi et al. presented the mathematical method for the theoretical prediction of the magnetic field distribution around a short crack (length 30 mm) [18]. Yuan et al. presented the two-step interpolation algorithm for the measurement of long and short cracks in the pipe string using the uniform alternating current field [19]. Leng et al. proposed the combined metal magnetic memory (MMM) and the ACFM technique for the detection and evaluation of critical weld joints in a jack-up offshore platform [20]. Rowshandel et al. presented the artificial neural network (ANN) for sizing the important subsurface section of the multiple cracks using ACFM [21]. These methods obtained good characteristic signals from the cracks without lift-off variations [22,23]. However, the characteristic signal and butterfly plot are cluttered when ACFM is used to identify tiny surface cracks in the rugged weld.

The aim of this paper is to find out tiny surface cracks in rugged welds. This paper is organized as follows: in Section 2, the insensitive signal to the lift-off variation is pointed out by simulations and experiments; in Section 3, the tiny crack is detected in the rugged weld and the signal gradient algorithm is presented to identify the tiny crack; and in Section 4, the conclusion and further work are given.

## 2. Insensitive Signal to Lift-off Variations

### 2.1. Simulation Model

In terms of the theory, the ***Bx*** is produced by the decrease of the current density in the depth direction of the crack. Thus, the ***Bx*** keeps an individual value (background signal) when a crack is not present. The ***Bz*** is produced by the deflection of the induced current field, thus the ***Bz*** is zero when a crack is not present. When the lift-off varies, the background signal changes—but the current field is still uniform (the current density value changes, but still no deflection). Thus, the ***Bx*** is easily affected by the lift-off variations and the ***Bz*** is insensitive to lift-off variations.

**Figure 3:**
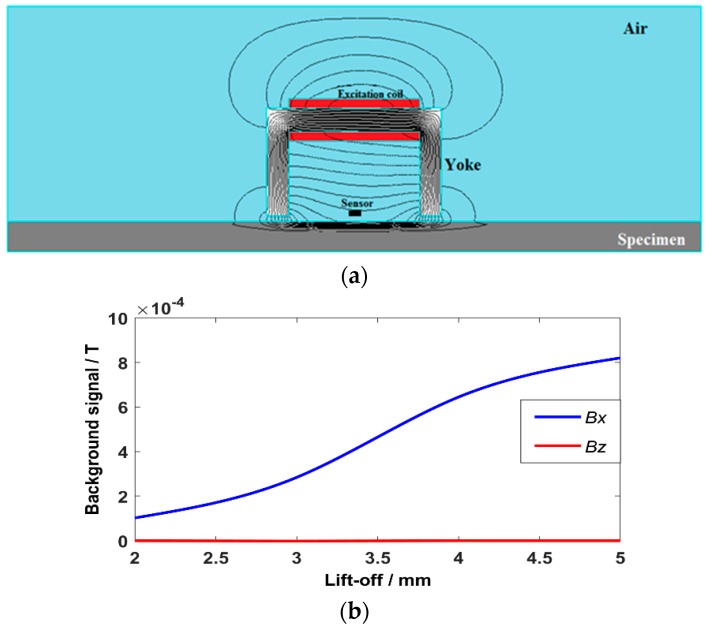
A 2D simulation model of ACFM probe. (**a**) Distribution of magnetic field; (**b**) background signal with different lift-off.

The 2D simulation model of the ACFM probe was set up as shown in Figure 3a. In the model, the frequency is 2 KHz and the current amplitude is 50 mA [24]. The material of the yoke is Mn-Zn ferrite and the specimen is a steel plate. The relative permeability of the yoke is 2000. The conductivity and relative permeability of the specimen are 5 × 10^6^ S/m and 200, respectively. Because steel permeability is much greater than air permeability, the magnetic line of force propagates from the U-shaped yoke to the steel. Meanwhile, due to the skin effect, most of the magnetic line of force gathers in the thin surface of the steel. The magnetic field produces a uniform current field in the center of the specimen [25,26]. 

When the lift-off increases, more magnetic field leaks into the air. Thus, the background signal of the ***Bx*** increases when the lift-off goes up. While there is no magnetic field in the Z direction, the ***Bz*** keeps zero because of the uniform current field, as shown in Figure 3b. We can make a conclusion that the ***Bx*** is easily affected by the lift-off variations and the ***Bz*** is insensitive to the lift-off variations.

### 2.2. System Set Up

**Figure 4:**
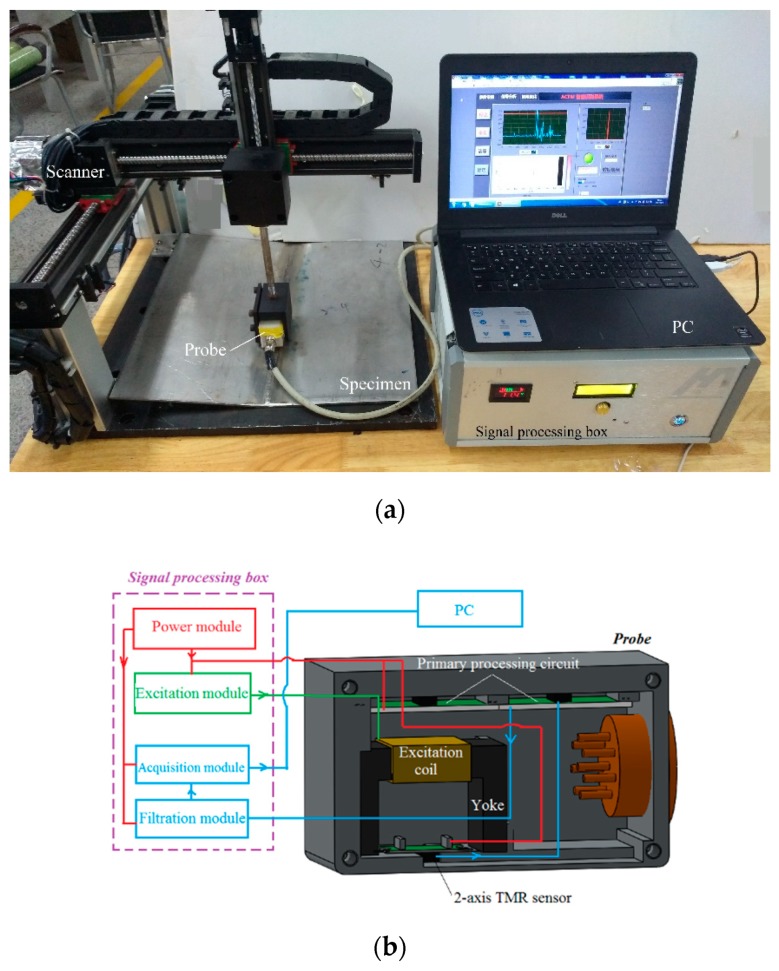
ACFM system. (**a**) Photo of the system; (**b**) probe and signal processing module.

The ACFM testing system is shown in Figure 4a. The system consists of three main parts: a probe, a signal processing box, and a 3-axis scanner. The probe includes an excitation coil, a MnZn-ferrite yoke, a detection sensor, and a primary processing circuit, as shown in Figure 4b. The excitation coil is wound on the MnZn-ferrite yoke with 500 turn enameled wires whose diameter is 0.15 mm. The detection sensor is a 2-axis tunnel magneto resistance (TMR) sensor that is set at the bottom center of the yoke [27,28]. The 2-axis TMR sensor (Type: TMR2303, Capacity: ±80 Oe, Sensitivity: 3 mV/Oe, made by MULTI DIMENSION, China), is used to measure the ***Bx*** and ***Bz*** at the same time. The ***Bx*** and ***Bz*** are amplified 50 times and 100 times, respectively, by the primary processing circuit. The thickness of the probe shell bottom is 1 mm to keep the lift-off of the sensor 1 mm. The probe is installed on a 3-axis scanner which is controlled by the programmable logic controller (PLC).

The signal processing box includes a power module, an excitation module, a filtration module, and an acquisition module, as shown in Figure 4b. The power module provides the power source to keep the modules running for more than six hours. The excitation module produces a sinusoidal signal, whose frequency is 2 KHz and amplitude is 10 V. The sinusoidal signal is loaded on the excitation coil in the probe. The ***Bx*** and ***Bz*** are filtered by the filtration module using a low-pass filter whose cut-off frequency is 20 KHz. The ***Bx*** and ***Bz*** are gathered by the acquisition module and transmitted to the personal computer (PC) for the signal processing and analyzing software.

### 2.3. Lift-Off Variation Experiments

**Figure 5:**
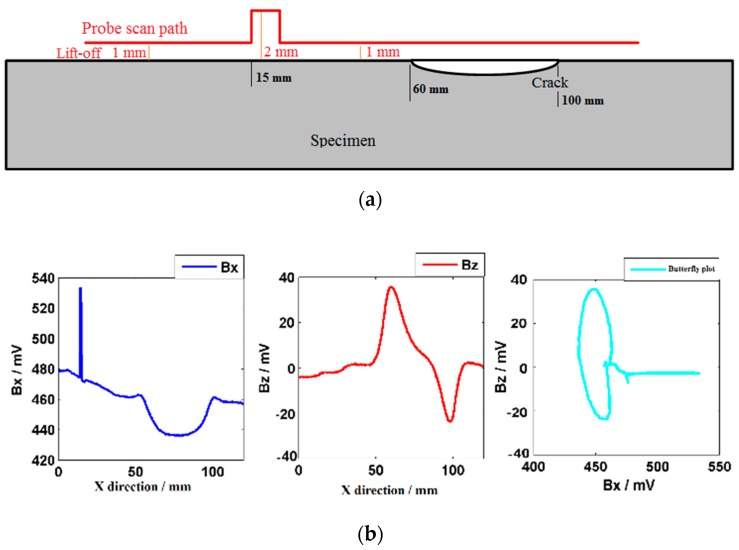
Testing results when lift-off upward. (**a**) Probe scan path; (**b**) ***Bx***, ***Bz***, and butterfly plot.

To verify the insensitive signal to lift-off variations, the probe is driven up and down above the specimen by the scanner. The first specimen is a mild steel plate with a semi-elliptical artificial crack (length: 50 mm, width: 0.5 mm and depth: 5 mm). There is no weld on the plate and the surface is flat. In the lift-off upward testing experiments, the probe is driven by the scanner to scan along the artificial crack with a lift-off of 1 mm at a speed of 2 mm/s. The probe is raised up 1 mm by the scanner and then dropped after 2 s, as shown in Figure 5a.

As shown in Figure 5b, the ***Bx*** shows a trough in the center of the crack, while the ***Bz*** shows opposite peaks at the tips of the crack. The characteristic signals are consistent with ACFM theory. When the lift-off is upward, the ***Bx*** shows a maximum peak and the ***Bz*** remains the same. This is because the background signal increases when the lift-off goes up. Meanwhile, the induced current field is still uniform and the ***Bz*** almost remains the same. There are obvious loops in the butterfly plot for the identification of a crack in the flat specimen. The ***Bx*** distorted value caused by the 1 mm lift-off variation is much larger than that caused by the crack, which produces an interference signal in the butterfly plot.

**Figure 6:**
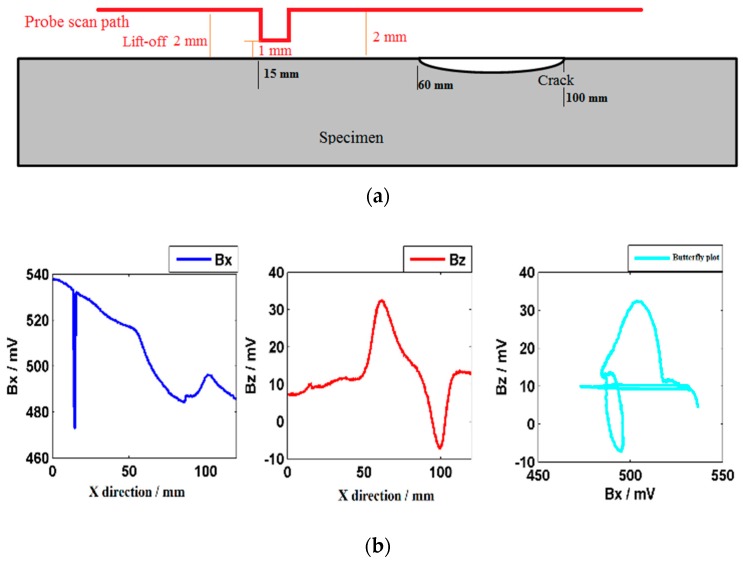
Testing results when probe downward. (**a**) Probe scan path; (**b**) ***Bx***, ***Bz***, and butterfly plot.

In the lift-off downward testing experiments, the probe was lowered down 1 mm by the scanner and then raised after 2 s, as shown in Figure 6a. When the lift-off is downward, the ***Bx*** shows a minimum trough and the ***Bz*** almost remains the same, as shown in Figure 6b. This is because the background signal decreases when the lift-off goes down. In the same way, the crack can be identified by the butterfly plot. The lift-off downward also produces an obvious interference signal in the butterfly plot.

**Figure 7:**
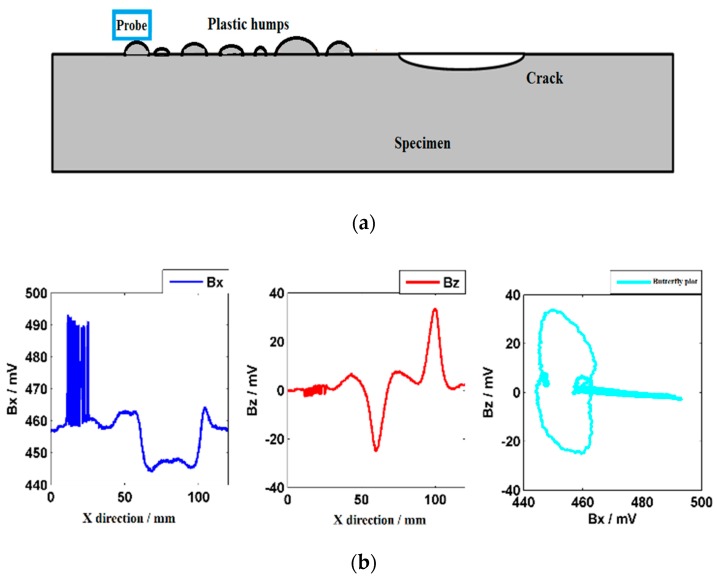
Testing results when lift-off varies seriously. (**a**) Probe scan path; (**b**) ***Bx***, ***Bz***, and butterfly plot.

To simulate the uneven surface of a weld, plastic humps were set on the surface of the specimen in front of the crack, as shown in Figure 7a. When the probe scans the humps, the lift-off varies seriously from 1 mm to 4 mm. The characteristic signals are shown in Figure 7b. The ***Bx*** shows hash signals, while the ***Bz*** varies slightly in the hump area. The results show that the ***Bx*** is affected seriously by the lift-off variations and the ***Bz*** is insensitive to the lift-off variations. The ***Bz*** can be set as the insensitive signal to identify the tiny surface crack in the weld.

## 3. Signal Gradient Algorithm

### 3.1. Detection of Tiny Cracks in the Weld and the HAZ

**Figure 8:**
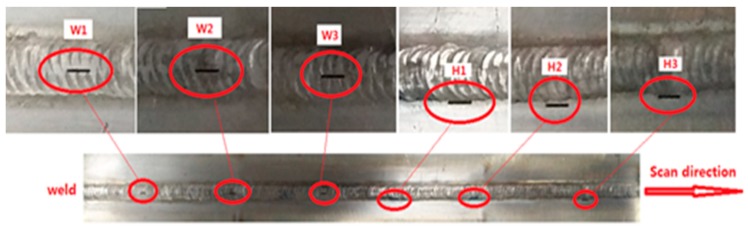
The second specimen with different depth cracks.

The second specimen is a plate with one weld, as shown in Figure 8. The material of the specimen is the same as the first specimen. The thickness of the plate is 4 mm. The width of the weld is 10 mm and the height of the weld reinforcement is 2 mm. (The distance between the weld and the HAZ is 2 mm.) The weld ripples are rugged and the thickness of the ripples range from 0.1 mm to 1 mm. Thus, when the ACFM probe scans along the weld, the lift-off of the probe varies from 0.1 mm to 1 mm.

There are three tiny surface cracks in the weld with the same length (4 mm) and different depths (3.5 mm, 3.0 mm and 2.5 mm). There are other 3 cracks with identical length of 4 mm and the same depths (W1, W2, and W3) in the HAZ. The depths of the cracks are given in Table 1.

**Figure 9:**
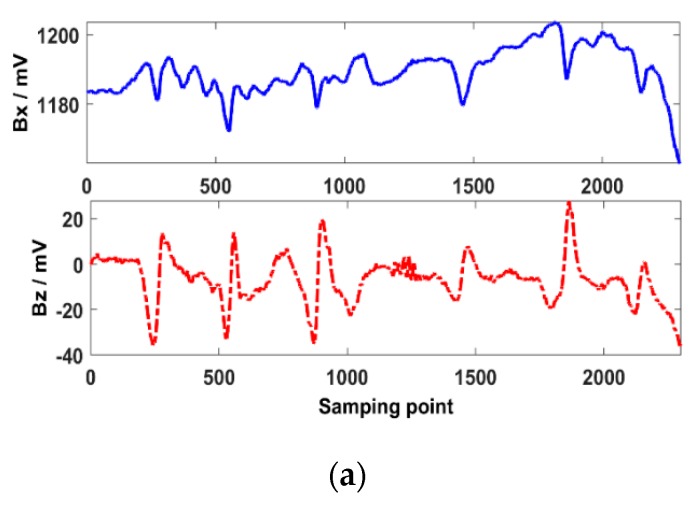

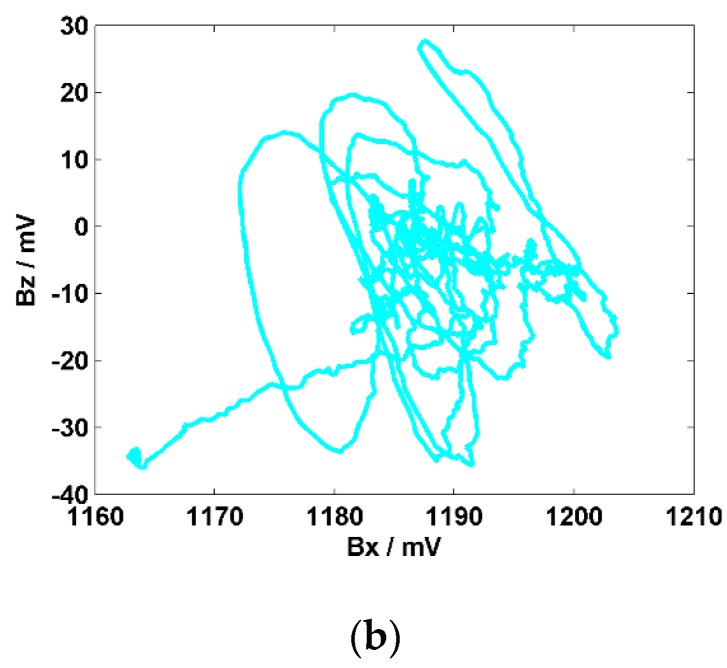
Characteristic signals of different depth cracks in the weld and the HAZ. (**a**) ***Bx*** and ***Bz***; (**b**) butterfly plot.

The probe is driven by the scanner to scan the weld from W1 to H3 at a speed of 5 mm/s. The probe can shake up and down with the ripples. The testing results are shown in Figure 9a. The ***Bx*** is chaotic because there are many interference signals caused by the lift-off variations. Thus, the cracks cannot be identified clearly by the ***Bx*** signal. There are six characteristic signals in the ***Bz*** when a crack is present. The SNR of the ***Bz*** is much better than that of the ***Bx*,** because the ***Bz*** is insensitive to the lift-off variations. As shown in Figure 9b, the butterfly plot is irregular, which cannot be used to identify the 6 tiny cracks.

**Figure 10:**
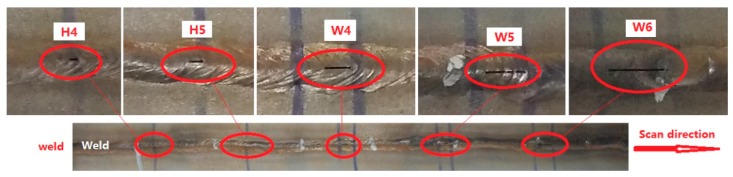
The third specimen with different length cracks.

The third specimen is shown in Figure 10. There are two different length cracks in the HAZ and three different length cracks in the weld. The cracks are at the same depth (3.0 mm). The lengths of the cracks are shown in Table 2. 

**Figure 11:**
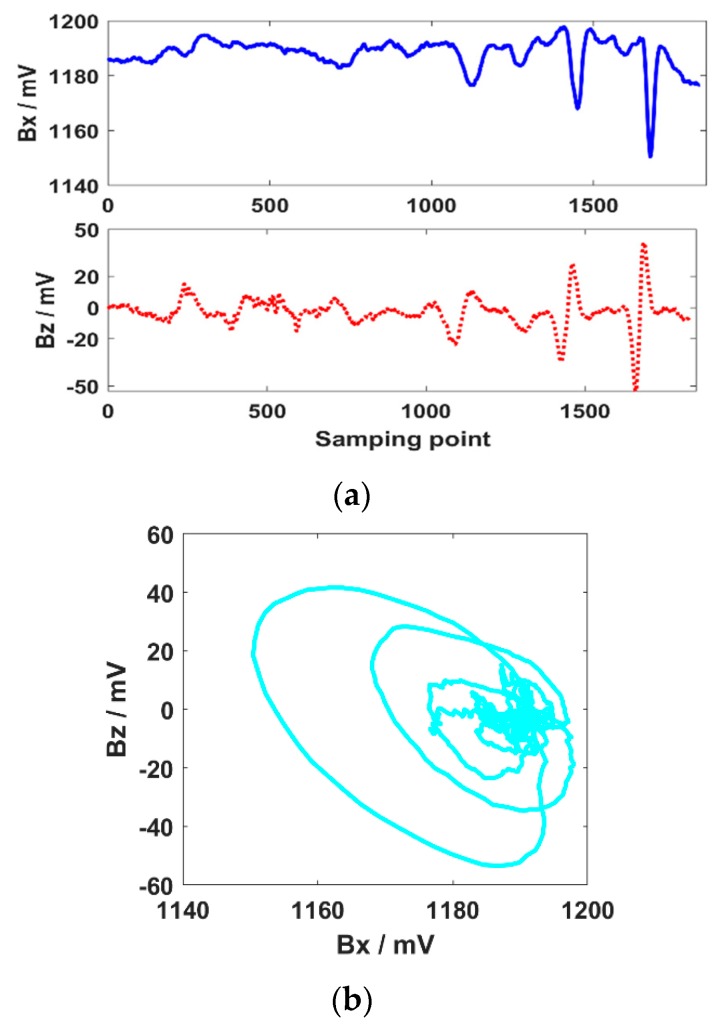
Characteristic signals of different length cracks in the weld and the HAZ. (**a**) Bx and Bz; (**b**) butterfly plot.

The weld was scanned at the same speed, and the testing results are shown in Figure 11. The ***Bx*** shows two troughs, and the ***Bz*** shows two troughs and peaks at the W5 and W6 cracks. The characteristics of the ***Bx*** and ***Bz*** are incomplete at the W4 crack. For the H4 and H5 cracks, there is one response in the ***Bx***, while the ***Bz*** shows slight perturbations because of the very short length of the crack. As shown in Figure 11b, there are two obvious loops in the butterfly plot, which can be used to identify the last two longer cracks in the weld. Other cracks cannot be identified effectively by the irregular tracks in the butterfly plot.

### 3.2. Signal Gradient Algorithm

The gradient method can find the amplitude change rate of the signal. The ***Bz*** changes are small when lift-off varies. Thus, the gradient of the ***Bz*** will be zero when a crack is not present. What’s more, there are opposite peaks at the two ends of the crack in the ***Bz***. As a result, a slight perturbation in the ***Bz*** caused by the crack is amplified many times by the gradient method. Thus, the gradient of the ***Bz*** will show an outstanding peak in the center of the crack. The SNR of the crack identification signal will be improved greatly in the near zero background signal.

**Figure 12:**
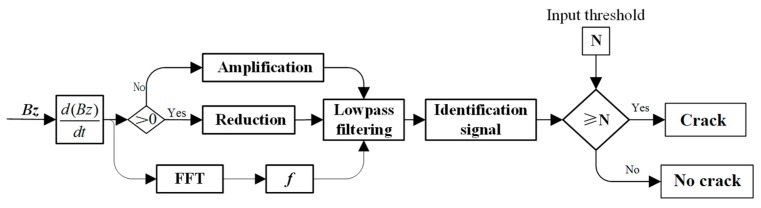
Signal gradient algorithm for the processes of the ***Bz***.

The signal gradient algorithm is presented to process the ***Bz*** by the following steps, as shown in Figure 12. First, the gradient of the ***Bz*** is calculated in real time. If a crack is not present, the value is near zero. When a crack is present, the gradient of the ***Bz*** will show some negative values and a major positive value. Then the negative values are decreased and the positive value is magnified. To make the curve smooth, the signal is processed by a 6-th order Butterworth low-pass filter. Thus, the identification signal is presented with a great positive peak and near zero value. The threshold is set beforehand to compare the identification signal with the threshold automatically. If the identification signal is greater than the threshold, the crack can be identified effectively because of the high SNR.

**Figure 13:**
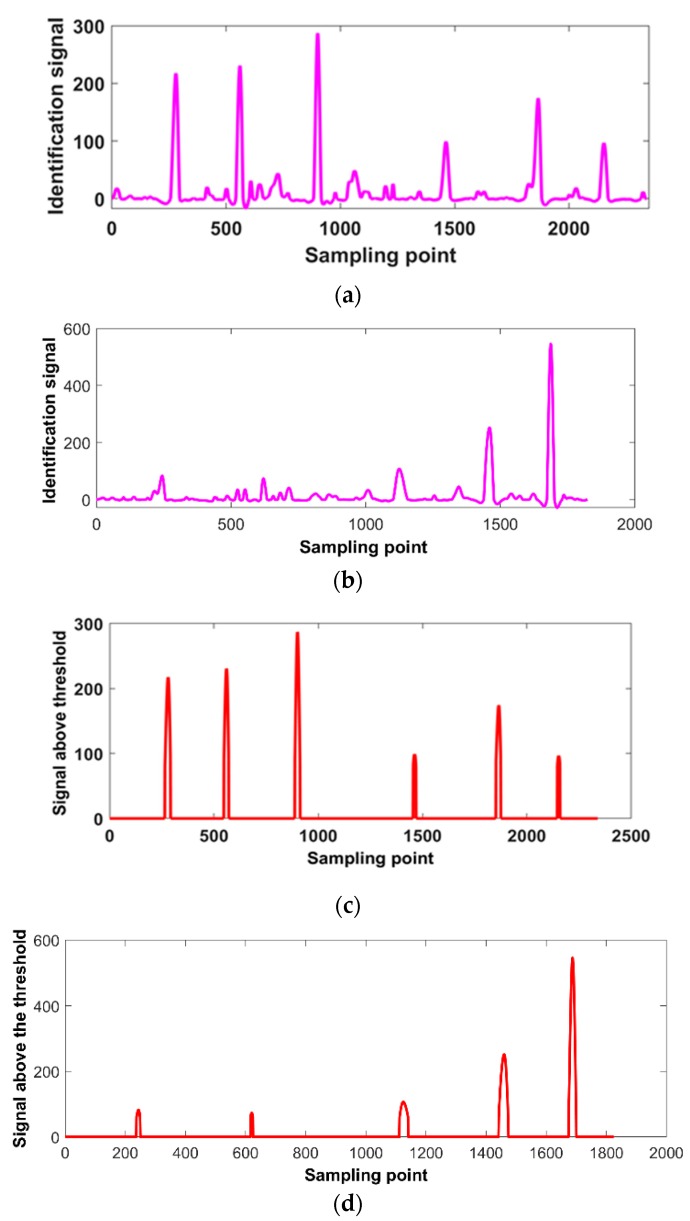
Identification of tiny cracks by signal gradient algorithm. (**a**) Identification signal of different depth cracks; (**b**) identification signal of different length cracks; (**c**) identification of different depth tiny cracks; (**d**) identification of different length tiny cracks.

The ***Bz*** signals of different depth cracks (from Figure 9a) and different length cracks (from Figure 11a) are processed by the signal gradient algorithm. The identification signals of the tiny surface cracks with lift-off variations in the weld and the HAZ are shown in Figure 13. As shown in Figure 13a, there are six obvious peaks in the identification signals caused by the different length cracks. Meanwhile, there are three obvious peaks caused by the three longer cracks in the weld and two slight peaks caused by the two shorter cracks in the HAZ, as shown in Figure 13b. To remove the background signal and identify the tiny cracks effectively in real time, the threshold can be set in advance using 1.5 times the maximum noise amplitude in the identification signal. In these experiments, the maximum noise amplitude was 53 and the threshold was set as 80. Thus, all six different depth cracks and five different length cracks can be identified effectively, as shown in Figure 13c,d, respectively.

### 3.3. Discussions

The SNR of the ***Bz*** signal is better than that of the ***Bx*** in the crack testing results, as shown in Figure 9a. This is because the ***Bx*** signals are greatly disturbed by the lift-off variations caused by the rugged weld ripples and reinforcements. The ***Bz*** is relatively stable because it is insensitive to the lift-off variations—thus, the ***Bz*** is used as the characteristic signal to identify the tiny crack with lift-off variations. As shown in Figure 11a, where the length of the crack is shorter than 4 mm, the distortion of the ***Bx*** and ***Bz*** decays seriously. This is because the disturbance of the induced current field declines sharply around the short cracks. For the 1 mm and 2 mm length cracks, the induced current field turns slightly and the magnetic field distorts weakly.

As shown in Figure 13a, the last three peaks are less than the first three peaks. This is because the last three cracks are in the HAZ and the distance between the probe and the HAZ is greater than the distance between the probe and the weld. Because the first three cracks are in the rugged weld, the background signal in the identification signal has a bit more clutter than that of the last three cracks. As shown in Figure 13b, the peaks of the three longer cracks are obvious and the peaks of first two shorter cracks are weaker. Because the length of the tiny cracks has a significant impact on the ***Bx*** and ***Bz*** signals, it is difficult to identify the shorter cracks (less than 2 mm) in the weld and the HAZ—the shorter cracks are easily covered by the noise signal. Overall, in these experiments the SNR of the identification signals must be relatively high to identify all the tiny cracks in the weld and the HAZ by threshold compared to the conventional butterfly plot. 

## 4. Conclusions

In this paper, a signal gradient algorithm was presented to identify tiny cracks in welds using the ACFM technique. The insensitive signal to the lift-off variations was pointed out by the simulations and experiments. The ACFM probe with a 2-axis TMR sensor and testing system were developed. The different depth and different length tiny surface cracks were detected in the rugged weld and the HAZ using an ACFM testing system. The results show that the ***Bz*** signal was the insensitive signal to the lift-off variations. The SNR of the crack response signal was greatly improved by the signal gradient algorithm. Thus, all the tiny surface cracks can be effectively identified in the weld and the HAZ by the signal gradient algorithm using the ACFM technique. Further work will focus on the identification of other type defects, evaluation of tiny defects in the weld, and the HAZ.

## Figures and Tables

**Table 1:** The depth of the cracks.

No.	W1	W2	W3	H1	H2	H3
Depth/mm	3.5	3.0	2.5	3.5	3.0	2.5

**Table 2:** The length of the cracks.

No.	H4	H5	W4	W5	W6
Length/mm	1.0	2.0	4.0	6.0	8.0
